# From classical dualistic antagonism to hormone synergy: potential of overlapping action of glucagon, insulin and GLP-1 for the treatment of diabesity

**DOI:** 10.1530/EC-23-0529

**Published:** 2024-04-25

**Authors:** Svjatoslavs Kistkins, Othmar Moser, Vitālijs Ankudovičs, Dmitrijs Blizņuks, Timurs Mihailovs, Sergejs Lobanovs, Harald Sourij, Andreas F H Pfeiffer, Valdis Pīrāgs

**Affiliations:** 1 Pauls Stradiņš Clinical University Hospital, Riga, Latvia; 2 Division of Exercise Physiology and Metabolism, Institute of Sport Science, University of Bayreuth, Bayreuth, Germany; 3 Institute of Smart Computing Technologies, Riga Technical University, Riga, Latvia; 4 Trials Unit for Interdisciplinary Metabolic Medicine, Division of Endocrinology and Diabetolgoy, Medical University of Graz, Graz, Austria; 5 Department of Endocrinology and Metabolic Medicine, Campus Benjamin Franklin, Charité University Medicine, Hindenburgdamm, Berlin, Germany; 6 Faculty of Medicine, University of Latvia, Riga, Latvia

**Keywords:** glucagon, insulin, GLP-1, diabesity, treatment

## Abstract

The increasing prevalence of ‘diabesity’, a combination of type 2 diabetes and obesity, poses a significant global health challenge. Unhealthy lifestyle factors, including poor diet, sedentary behaviour, and high stress levels, combined with genetic and epigenetic factors, contribute to the diabesity epidemic. Diabesity leads to various significant complications such as cardiovascular diseases, stroke, and certain cancers. Incretin-based therapies, such as GLP-1 receptor agonists and dual hormone therapies, have shown promising results in improving glycaemic control and inducing weight loss. However, these therapies also come with certain disadvantages, including potential withdrawal effects. This review aims to provide insights into the cross-interactions of insulin, glucagon, and GLP-1, revealing the complex hormonal dynamics during fasting and postprandial states, impacting glucose homeostasis, energy expenditure, and other metabolic functions. Understanding these hormonal interactions may offer novel hypotheses in the development of ‘anti-diabesity’ treatment strategies. The article also explores the question of the antagonism of insulin and glucagon, providing insights into the potential synergy and hormonal overlaps between these hormones.

## Introduction: diabesity as a growing public health problem

The epidemic of ‘diabesity’, a co-syndrome of type 2 diabetes (T2D) and obesity, is predicted to be one of the largest healthcare issues in human history (1). In 2016, over 650 million people were obese (2), while approximately 537 million adults are living with diabetes worldwide (3), with numbers expected to increase over the coming years. In people with T2D, obesity is estimated to increase the costs by almost 30% as compared to normal weight people with diabetes (4). With mounting cases of diabesity, especially among geriatric patients, the obesity and diabetes treatment markets are estimated to witness significant growth. The diabesity pandemic is spurred by rapidly changing lifestyles, which include unhealthy dietary habits, sedentary behaviour, and increasing stress levels, interplaying with genetic and epigenetic factors (5). The intertwining of these two conditions amplifies the risk of various health complications such as cardiovascular diseases, stroke, and certain types of cancer (6). From a pathophysiological point of view, an excessive white adipose tissue mass is linked to increased insulin resistance, aggravating age-dependent risk of T2D and atherosclerosis (7). While this likely depends on several mechanisms, data from both *in vitro* and clinical trials suggest that impaired insulin sensitivity in white adipose tissue plays an early role in dysregulation of adipocyte metabolism and whole-body insulin resistance (8, 9). This article aims to not only observe novel strategies in the treatment of diabesity but also to describe the unique qualities of previously unseen hormone cross links between insulin, glucagon, and GLP-1.

### Overview of perspectives in the combinatory use of incretin-based therapies in diabesity and physiological nature of hormonal crosstalk

The primary objective of treating diabetes in particular novel pharmacological options leading to significant weight loss is to enhance insulin sensitivity and beta-cell function and subsequently glycaemic control. GLP-1 receptor agonists and dual hormone therapies have exhibited promising outcomes in those with T2D and obesity. Tirzepatide, a dual GLP-1 and GIP receptor agonist, has demonstrated to improve glycaemia, to increase glucose-dependent insulin secretion, and to reduce significantly body weight. Tirzepatide (5 mg, 10 mg, 15 mg) was compared with semaglutide (1 mg) and it was shown that the body weight loss was more pronounced in the tirzepatide groups (−7.6 kg, −9.3 kg, −11.2 kg, respectively) compared to the semaglutide group (−5.7 kg). GLP-1/GIP/GCG receptor agonists besides GLP-1 and GIP also active the glucagon receptor potentially increasing energy expenditure as another mechanisms (10). Retatrutide, a member of this receptor agonist family has demonstrated within a phase 1 trial in people with T2D to reduce dose dependently HbA1c by up to 1.5% and body weight by up to 9 kg within 85 days of treatment (11). Recently, retatrutide was demonstrated in obese people to reduce body weight by up to 24.2% over 48 weeks (12). In addition, dual agonists, combining GLP1 and glucagon receptor activation are currently under development, of which survodutide (BI 456906) was shown to reduce body weight up to 15% in overweight/obese people over 46 weeks (13).

However, the physiological role of gut hormones is to predominantly work in a short, oscillatory manner rather than in a prolonged way. The secretion of GLP-1 and GIP from intestinal endocrine cells is crucial to reduce appetite when it is physiologically needed within the hormonal changes in postprandial state. Moreover, studies showed that the withdrawal of long-acting incretin-based therapies may significantly regain body weight (14).

An additional insulin supplementation may be needed in patients with insulin-deficient diabetes, which can further aggravate their body weight. Currently, the combination of insulin analogues and GLP-1 receptor agonists (e.g. insulin degludec with liraglutide) is available, but it primarily suppresses weight gain rather than promoting weight reduction. In the context of diabesity, it is crucial to highlight the relevance of the combination and interplay of insulin with other hormones, particularly for patients with a manifest insulin deficiency who may still require insulin even after achieving significant weight loss. This scenario is of particular significance for individuals with long-standing T2D who are already requiring insulin therapy. By incorporating a combination of insulin and other hormones or hormone receptor agonists, we can address the complex hormonal interactions and optimize metabolic and cardiovascular treatment outcomes in these individuals.

**Figure 1:**
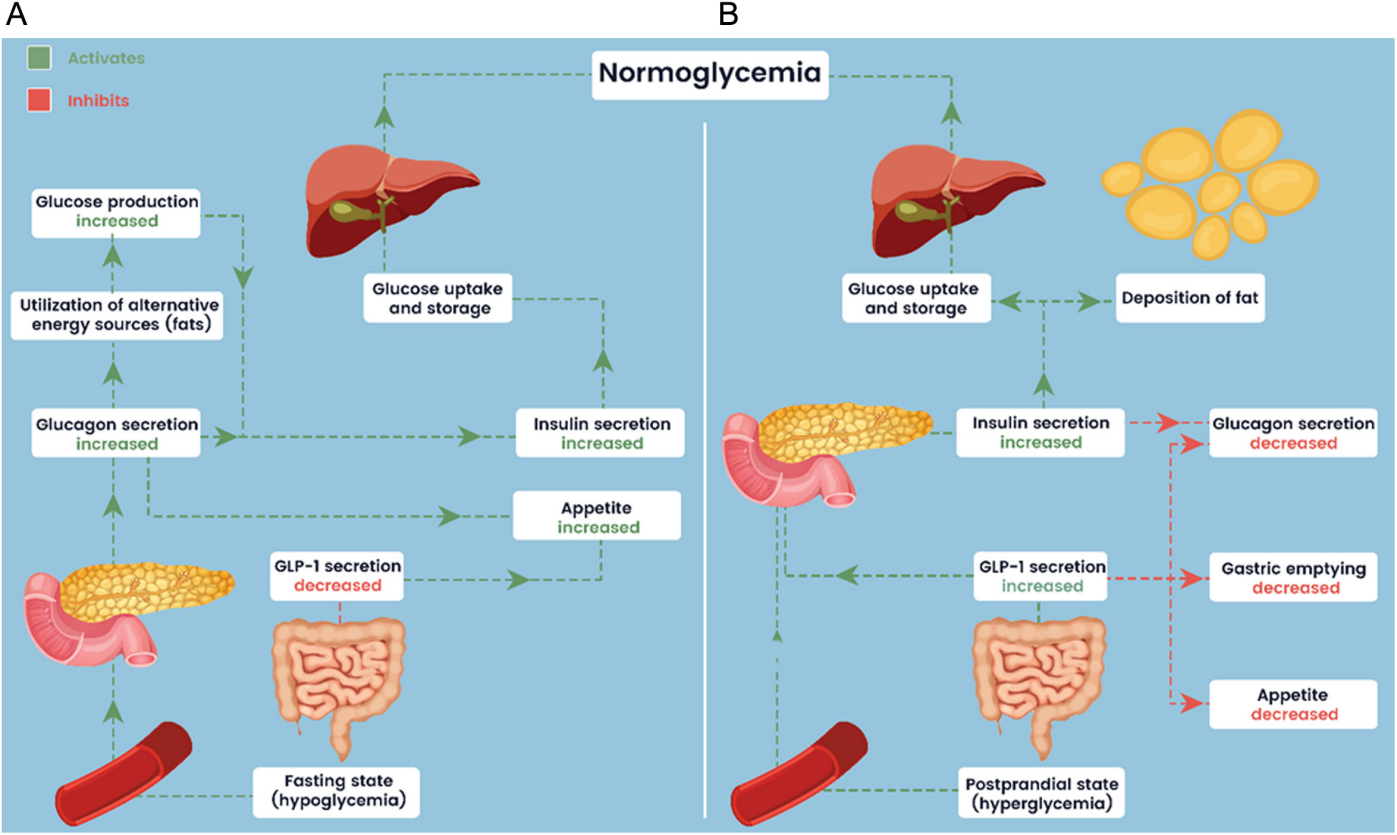
Fasting (A) and postprandial hormonal interactions. (B) In a state of hypoglycaemia, increased glucagon secretion prompts the utilization of glycogen and lipids, resulting in glucose and fatty acids production. Glucagon in the absence of GLP-1 increases appetite. Conversely, both glucagon and glucose stimulate insulin secretion, facilitating glucose uptake and storage in organs. This process primes the body for food intake and glucose replenishment (A). Following a meal, hyperglycaemia triggers secretion of GLP-1 and insulin. GLP-1 reduces gastric emptying and appetite. Both GLP-1 and insulin suppress glucagon secretion. This optimizes the rate of glucose deposition into the blood, enhancing insulin secretion to facilitate glucose uptake in organs and fat deposition (B).

An alternative strategy could involve incorporating glucagon or glucagon receptor agonists in conjunction with insulin and GLP-1 receptor agonists, targeting the complex interplay between these hormones and potentially offering benefits for both insulin-deficient diabetes and obesity management. Glucagon is known to promote energy expenditure via stimulation of lipolysis in adipocytes, hepatocytes, and cardiomyocytes and a proper use of the hormone may provide several benefits described below. As the prevalence of diabesity continues to rise, it is crucial to deepen our understanding of its pathophysiology, prevention strategies, and effective treatment interventions. It is particularly important for gaining in-depth knowledge of potential treatment strategies for diabesity by comparing the cross-interactions of incretins such as GLP-1 and pancreatic hormones − insulin and glucagon − during the caloric restriction with postprandial hormonal changes (Fig. 1). In a state of hypoglycaemia, increased glucagon secretion prompts the utilization of glycogen and lipids, resulting in glucose and fatty acids production. Glucagon in the absence of GLP-1 increases appetite. Conversely, both glucagon and glucose stimulate insulin secretion, facilitating glucose uptake and storage in organs. This process primes the body for food intake and glucose replenishment (Fig. 1A). Following a meal, hyperglycaemia triggers the secretion of GLP-1 and insulin. GLP-1 reduces gastric emptying and appetite. Both GLP-1 and insulin suppress glucagon secretion. This optimizes the rate of glucose deposition into the blood, enhancing insulin secretion to facilitate glucose uptake in organs and fat deposition (Fig. 1B).

During fasting both GLP-1 and insulin secretion decrease. Conversely, glucagon secretion increases, further maintaining insulin secretion. The main role of glucagon in this process is to stimulate utilization of glycogen and lipids, resulting in reduced body fat levels and maintaining normal blood sugar levels. On the other hand, high intake of proteins stimulates glucagon secretion, subsequently increasing insulin production. In this case increased insulin level stimulate amino acids uptake and glucose production using alternative sources of energy, helping maintain normoglycaemia and normal body weight. Increased intake of carbohydrates stimulates GLP-1 secretion and insulin secretion, both GLP-1 and insulin decrease glucagon secretion. In this scenario, increased insulin levels promote glucose uptake and storage, leading to weight gain and maintaining normal blood sugar levels.

#### Fasting hormonal interactions

**Figure 2:**
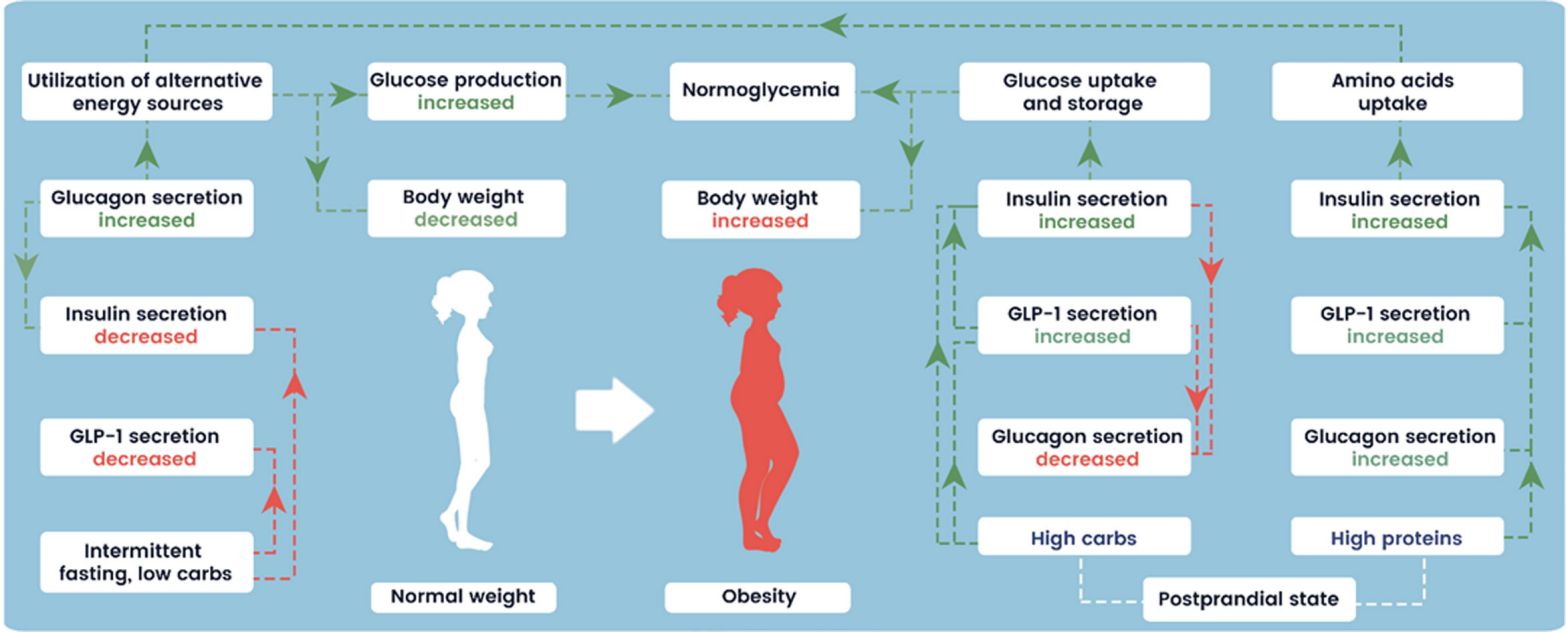
Glucose, GLP-1, insulin, and glucagon levels during fasting and postprandial state. During fasting both GLP-1 and insulin secretion decrease. Conversely, glucagon secretion increases, further maintaining insulin secretion. The main role of glucagon in this process is to stimulate utilization of glycogen and lipids, resulting in reduced body fat levels and maintaining normal blood sugar levels. On the other hand, high intake of proteins stimulates glucagon secretion, subsequently increasing insulin production. In this case, increased insulin levels stimulate amino acids uptake and glucose production using alternative sources of energy, helping maintain normoglycaemia and normal body weight. Increased intake of carbohydrates stimulates GLP-1 secretion and insulin secretion, both GLP-1 and insulin decrease glucagon secretion. In this scenario increased insulin levels promote glucose uptake and storage, leading to weight gain and maintaining normal blood sugar levels.

During caloric restriction (Fig. 2), the body undergoes complex hormonal changes to maintain glucose homeostasis. Glucagon, a secretion product of the pancreatic alpha cells, plays a vital role in this process. When blood glucose levels are low, glucagon secretion increases. Surprisingly, glucagon activates insulin secretion through G-protein-coupled receptors on beta cells even though insulin levels are generally low during fasting (15). Another hormone affected by fasting is GLP-1, produced by L cells in the intestines. In the fasting state, when nutrient availability is low, GLP-1 release is diminished.

GLP-1 and insulin secretion is primarily regulated by the presence of nutrients in the gastrointestinal tract, particularly glucose and fatty acids. Glucagon acts on its receptor, a G-protein coupled receptor (GPCR) expressed primarily in liver cells, stimulating glycogenolysis (breakdown of glycogen into glucose) and gluconeogenesis (synthesis of glucose from non-carbohydrate precursors). The decreased glucose uptake by peripheral tissues and increased lipolysis in adipose tissue, promoting the release of free fatty acids into circulation as an alternative energy source is observed while insulin levels are low. These metabolic adaptations help maintain glucose availability for vital organs, such as the brain, while also ensuring a steady supply of fatty acids for energy production. However, it is worth mentioning that low levels of insulin still persist in the bloodstream, providing basic redistribution functions. The intricate interplay between glucagon, insulin, and GLP-1 (Fig. 1ABM) during intermittent fasting allows the body to maintain glucose homeostasis and utilize alternative fuel sources to meet energy demands (16).

#### Postprandial hormonal interactions

In the postprandial state, a different coordinated interplay between insulin, glucagon, and GLP-1 is observed, highlighting the role of insulin in the process. In response to increased blood glucose levels, insulin secretion is triggered by direct glucose stimulation via GLUT2 channel or indirectly via GLP-1R mediated response (17). This amplification of insulin release helps to further lower blood glucose levels after a meal. Compared to fasting state, insulin and GLP-1 secretion in presence of local postprandial hyperglycaemia suppresses glucagon release (18, 19). As a result, the following peripheral glucagon receptor signalling is reduced. This suppression of glucagon prevents excessive hepatic glucose output and helps to maintain a steady-state blood glucose concentration. As a result, studies shows that low-to-moderate carbohydrate consumption has an increased energy expenditure (20) Regarding glucagon's actions, an essential aspect to consider is its involvement in amino acid metabolism. Glucagon is highly stimulated by amino acids and plays a regulatory role in the degradation of amino acids within the urea cycle and the alpha cell–liver axis (21). Furthermore, ureagenesis activates AMP-kinase, contributing to the energy expenditure induced by glucagon (22). Consequently, glucagon levels increase after meals, depending on the uptake of amino acids following food consumption (23). The rate of glucagon elevation varies depending on the protein source. Some proteins like casein cause minimal glucagon increase, while rapidly absorbed proteins elicit a substantial rise in glucagon levels. This triggers a compensatory insulin secretion, which is also observed in individuals with T2D.

At the same time, insulin acts on its receptor, promoting glucose uptake into insulin-sensitive tissues, such as skeletal muscle and adipose tissue, thereby reducing blood glucose levels. Insulin inhibits hepatic glucose production, suppressing glycogenolysis and gluconeogenesis. These actions ensure that excess glucose derived from the meal is efficiently stored and utilized, preventing postprandial hyperglycaemia. Additionally, GLP-1 slows down gastric emptying, which helps regulate the rate of nutrient absorption and prevents spikes in blood glucose levels. GLP-1 acts on its receptor, a GPCR found in various tissues, including the pancreas and brain. GLP-1 has been shown to increase satiety and reduce food intake, contributing to weight management and potentially preventing overeating. Data shows that high-protein diet increases energy expenditure, promoting body weight reduction (24).

In summary, glucagon secretion is increased during the fasting state, while GLP-1 and insulin secretion are low. Glucagon in the presence of low insulin levels stimulates glucose production, to promote the utilization of alternative energy sources. The rapid and pulsatile interplay of glucagon and insulin in the fasting state has been elaborated by Nauck and Meier in healthy people and in prediabetes or diabetes (25, 26). Glucagon is suppressed by hyperglycaemia in healthy people while this is lost in T2D. One possibility is that the liver becomes tolerant to the hyperglycaemic effects of glucagon in T2D upon the use of long-acting glucagon agonists. Prolonged fasting also leads to a switch from glucagon to cortisol to maintain hepatic glucose production.

In the postprandial state, it is worth to divide physiology of carbohydrate-rich and protein-based food: glucagon secretion is suppressed by carbohydrate-rich food and insulin/GLP-1 dual secretion, while protein-based food stimulates both glucagon and insulin/GLP-1 triple secretion. In carbohydrates-rich food, high insulin level in the presence of low glucagon level facilitates glucose uptake and storage in insulin-sensitive tissues, while GLP-1 enhances insulin secretion and exerts additional effects to regulate glucose homeostasis. Notably, a high protein intake has been shown to improve various aspects of liver function and reduce liver fat content in individuals with T2D (22, 27, 28).

In the case of diabesity additional insulin administration impacts not only insulin resistance but also body weight by direct and indirect effects. However, as we mentioned before, patients with insulin-deficient diabetes require additional insulin therapy, promoting imitation of hormonal interaction during postprandial state. As a result, in case of obesity, insulin and GLP-1RA inhibits physiological glucagon functions while GLP-1RA stimulates insulin secretion disbalancing the equilibration of fasting/postprandial state. At the same time, the inhibition of glucagon after oral glucose or i.v. glucose differs in T2D (29, 30). While the inhibition after oral glucose is reduced or lost, i.v. glucose powerfully inhibits glucagon release in T2D. The mechanism appears to be intra-islet and may involve somatostatin (18) but other mechanisms were also proposed (31). Regarding the effects on appetite and insulin sensitivity, there are some papers claiming central effects of glucagon in mice which may differ from effects in humans (32).

Many studies suggest that caloric restriction, especially carbohydrate restriction, promotes not only bodyweight reduction but also insulin sensitivity. In this case, the physiology of caloric restriction may help to promote insulin sensitivity. Previously Habegger’s team reported that there was an unforeseen improvement in insulin function among db/db mice after receiving 7-day therapy with glucagon receptor agonist (33). In this case, a potential additional infusion of glucagon will promote not only an enhancement in insulin sensitivity but also energy expenditure. A crucial paper was published by Shulman's team in 2020 showing that glucagon acts through the INSP3R1 pathway to induce hepatic lipolysis and enhance mitochondrial ATP-production (34). This explains why amino acids strongly reduce liver fat via glucagon release. A similar effect may be expected for pharmacological glucagon agonists. This also explains the improvements of ATP:ADP ratio in the liver. Certainly, in a case of diabesity, the use of a short-acting glucagon infusion only will aggravate hyperglycaemia; thus, a proper additional insulin therapy would be crucial to maintain euglycaemia and accelerate energy expenditure in patients with insulin-deficient diabetes. However, hyperglycaemia due to glucagon was not observed postprandially in T2D but only in the fasting state using glucagon antagonists (35). The action of glucagon may be modified by co-agonists: clinical trials will answer this question in the future.

It is worth mentioning that the hypoaminoacidaemia elicited by glucagon may play a role in energy expenditure and may lead to loss of lean mass in glucagon co-agonists, particularly if there is no compensatory increase in protein intake (36, 37, 38). However, the studies only looked for loss of fat mass and body weight and somewhat neglected this potential negative side effect, since old people are often confronted with muscle loss and sarcopenia. The metabolic problem is that glucagon appears to elicit a persistent gluconeogenic effect in T2D which was quite clearly shown by the use of glucagon antagonists – which also increased liver fat and circulating fat as may be expected (35, 39). Overall, there is still a lack of understanding of the full beneficial or potentially deleterious impact of glucagon, which needs to be further elucidated in currently ongoing larger glucagon agonist trials (40, 41). Insulin also regulates protein and amino acid metabolism. In this case, an anabolic effect of insulin infusion may potentially be useful for the increase of muscle weight. Table 1 summarizes the impact of non-pharmacological and pharmacological treatment of mono and dual agonists, raising the fundamental question – will simultaneous administration of glucagon and insulin antagonize each other or might they be used in a synergistical way?.

**Table 1:** Different effects of non-pharmacological and pharmacological therapies on hormone levels, energy expenditure, and body weight for people with type 2 diabetes and/or obesity.

	Insulin		Glucagon		GLP-1		Energy expenditure	Body weight
	Total	Endo	Total	Endo	Total	Endo		
Intermittent fasting	Reduces (15)	Reduces (15)	Increases (15)	Increases (15)	Reduces (49)	Reduces (49)	Reduces (16)	Reduce (16)
High-carbohydrate diet	Increases (17)	Increases (17)	Reduces (18, 19)	Reduces (18, 19)	Increases (17, 49)	Increases (17, 49)	Reduces (20)	Increases (20)
High-protein diet	Increases (23)	Increases (23)	Increases (23)	Increases (23)	Increases (17)	Increases (17, 49)	Increases (24)	Reduces (24)
Insulin administration	Increases	Reduces (50)	Reduces (18)	Reduces (18)	Increases (50)	Increases (50)	No impact	Increases
GLP-1 RA administration	Increases (45) (hyperglucaemia)	Increases (45)	Reduces (45)	Reduces (45)	Increases (45)	No data	No impact (45)	Reduces (10)
Insulin + GLP-1 RA administration	Increases	No data	Reduces (18, 19)	Reduces (18, 19)	Increases	No data	No impact (45)	No data
Glucagon + GLP-1	Increases (45)	Increases (45)	Increases (45)	No data	Increases (45)	No data	Increases (44) (within 24 h)	Reduces (43)
Insulin + glucagon	Increases	No data	Increases	No data	No data	No data	No data	No data
Insulin + glucagon + GLP-1RA	No data	No data	No data	No data	No data	No data	No data	No data

Dual hormone therapies, such as cotadutide, a GLP-1 receptor and glucagon receptor co-agonist, have also been found to enhance glucose metabolism and weight control. These dual receptor agonists display a given receptor affinity for GLP-1 and glucagon, which cannot be changed. A recent study showed a reduction in body weight and a normalization in glycaemia, infusing both hormones simultaneously (42). However, this specific dual treatment might not be suitable for the majority, especially when exogenous insulin administration is required to maintain proper glycaemia. Moreover, the prolonged continuous administration of the glucagon may neglect the effect of the hormonal synergy, escaping any increase of energy expenditure (43). Previously, glucose homeostasis and resting metabolic rate/energy expenditure were observed to be elevated in those treated with dual hormone therapy, comprising a GLP-1 analogue and glucagon. Despite the effect of long-term hormonal intervention, the mean difference between the placebo and the short-term dual infusion of glucagon and GLP-1 was 146 kcal/day (44). The main implication was that GLP-1-stimulated insulin secretion, thereby reducing the hyperglycaemic effect of glucagon. These data also revealed no differences in calories between using glucagon infusion and a combination of glucagon and GLP-1-stimulated insulin secretion, raising the question of whether insulin and glucagon should still be considered pure antagonists.

### Insulin and glucagon – forgotten synergists in regulation of energy metabolism?

It is well known that insulin and glucagon are interpreted as two antagonistic hormones in a clear inverse relationship. However, an article that was published in June 2022 by Habegger (45) discusses a novel insight in glucagon and insulin pathway crosstalk. In Habegger’s review, he highlights the role of glucagon in enhancement of insulin resistance in mice model regulating the relative levels of hepatocyte IRS1:IRS2 ratios through the induction of Ppargc1a as a transcriptional regulator. The review exposes many unanswered scientific questions about the hormone synergy. Despite the fact that insulin is a physiological antagonist of glucagon and classically known to promote weight gain, the results of a clinical trial (44) indirectly neglect the current existing dogma. No statistical differences in energy expenditure between glucagon infusion only and a combination of glucagon and GLP-1 were observed in the study. The mean difference between placebo and each hormonal intervention was 146–147 kcal/day. At the same time, GLP-1-stimulated insulin secretion during glucagon-induced hyperglycaemia, suggesting that insulin might not change the energy expenditure caused by glucagon but normalizes glucose level:

GLP-1 vs placebo → 2.4 kcal/day* (−63.49; 68.43) (no insulin secretion)

Glucagon vs placebo → 146.99 kcal/day* (83.75; 210.24)(insulin secretion ↑)

Glucagon + GLP-1 vs placebo → 146.26 kcal/day* (82.58; 209.95)(insulin secretion ↑↑↑)

Glucagon + insulin vs placebo → **?**

*Tan *et al.* (2013) (44).

Furthermore, in 2018, Pedersen *et al.* investigated the impact of the dual therapy of long-acting glucagon analogue (LAG) and insulin on rats’ body weight (46). A decrease of body weight during dual infusion insulin in combination with LAG was observed, while insulin monotherapy increased the body weight.

## Overlaps and specific action of each hormone

**Figure 3:**
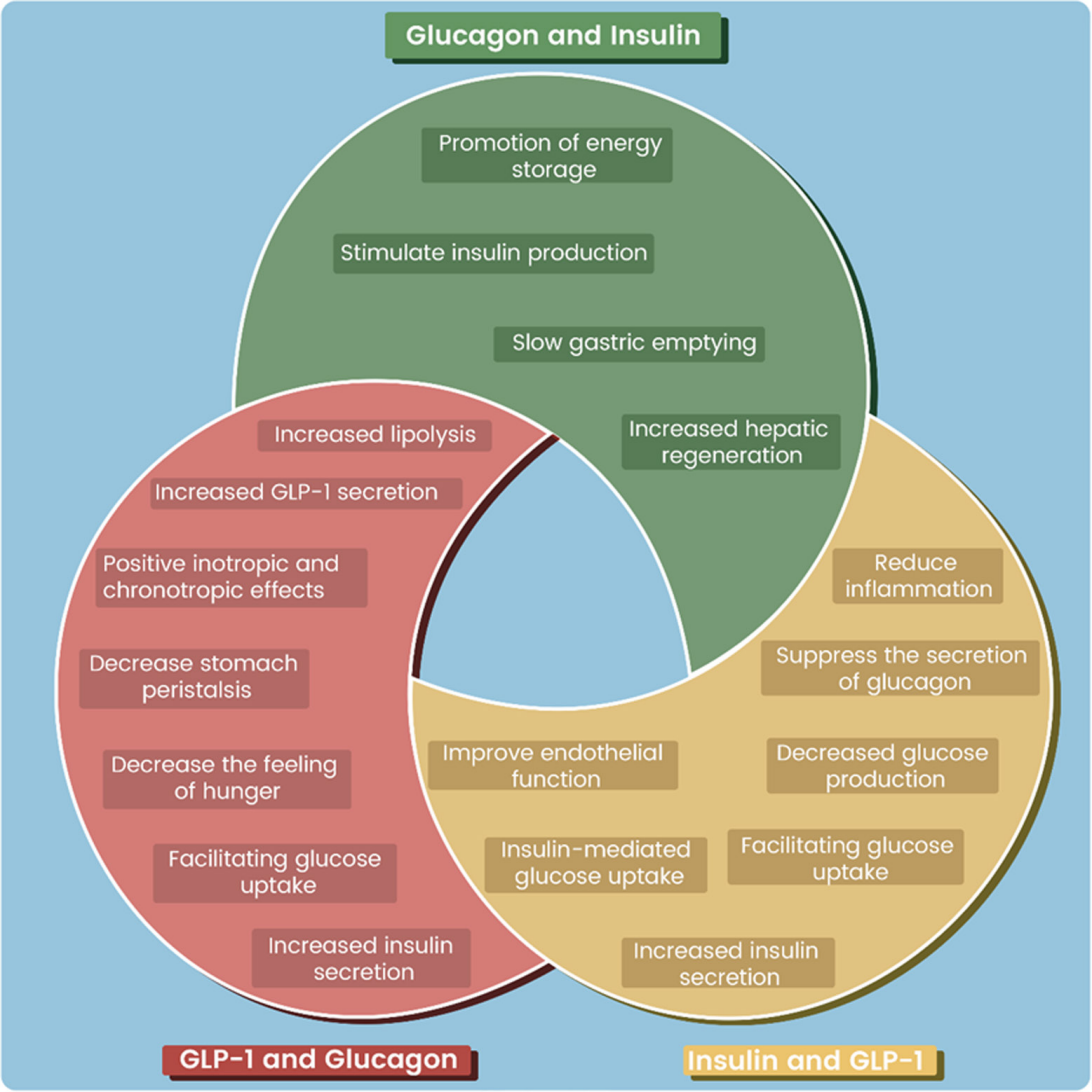
Triple hormone therapy action overlap. This figure vividly illustrates the overlapping actions of three key hormones: insulin, GLP-1, and glucagon. It is evident that all three hormones exhibit numerous overlapping actions. Insulin and GLP-1 improve endothelial function, promote glucose uptake, increase insulin production, decrease glucagon secretion, lower glucose production, and reduce inflammation. Similarly, both glucagon and insulin promote energy storage, stimulate insulin production, delay gastric emptying, and improve hepatic regeneration. Additionally, GLP-1 and glucagon boost insulin secretion, facilitate glucose uptake, decrease feeling of hunger, decrease stomach peristalsis, induce positive inotropic and chronotropic effects, increase GLP-1 secretion, and promote lipolysis.

In the following section, further interplay between insulin, glucagon, and GLP-1 will be discussed, exploring hormone dual overlapping functions and potential therapeutic implications (Figure 3). By unravelling the complexities of these hormonal interactions, we aim to gain a deeper understanding of how to optimize dual hormone therapies for the effective management of metabolic disorders and weight control and provide a new approach in combination of three hormones.

### Overlap in insulin and GLP-1 action

GLP-1 can enhance insulin signalling in peripheral tissues. GLP-1 has been shown to stimulate the translocation of GLUT4 glucose transporters in adipocytes and muscle cells, enhancing insulin-mediated glucose uptake (47). Conversely, insulin has been shown to upregulate the expression of GLP-1 receptors in pancreatic beta cells. This positive feedback mechanism can enhance the GLP-1-induced insulin secretion, further promoting glucose regulation (17).

There is also overlap in hormone synthesis. GLP-1 is secreted by nutrient induces signalling (48). Research has shown that insulin can stimulate the release of GLP-1 from the gut. This is thought to occur through the activation of insulin receptors in the gut, which then trigger the release of GLP-1. When L cells become resistant to insulin, it can lead to a decrease in the release of GLP-1 in response to various stimuli (49). GLP-1 receptor agonists promote insulin secretion in a glucose-dependent manner. Both insulin and GLP-1 RAs suppress the secretion of glucagon, a hormone that raises blood glucose levels. Insulin inhibits glucagon release (18), while GLP-1 RAs act on pancreatic alpha cells to suppress glucagon secretion (19).

Furthermore, there is overlap in the effect on target organs. Research has shown that when insulin and GLP-1 are both present, they can have a synergistic effect on lowering blood glucose levels. Insulin lowers blood glucose levels by facilitating glucose uptake into cells and inhibiting glucose production by the liver (50). Additionally, both insulin and GLP-1 RAs reduce hepatic glucose production. Insulin achieves this by inhibiting gluconeogenesis and glycogenolysis in the liver (51), while GLP-1 RAs indirectly suppress hepatic glucose production through their inhibitory effect on glucagon secretion (52). Insulin reduces glucotoxicity and lipotoxicity in beta cells (53), while GLP-1 RAs potentially impacts beta-cell function and survival (54). Insulin and GLP-1 RAs also provide some vascular benefits. Insulin has been shown to improve endothelial function (55), while GLP-1 RAs exhibit anti-inflammatory properties that help reduce vascular inflammation and atherosclerosis (56). GLP-1 RAs can also improve endothelial function by increasing nitric oxide (NO) production and promoting vasodilation (57). Furthermore, some studies have demonstrated that GLP-1 RAs can reduce macrophage foam cell formation, a critical event in the development of atherosclerotic plaques (58).

### Overlap in GLP-1 and glucagon action

Both glucagon and GLP-1 are derived from the proglucagon precursor molecule. Proglucagon is synthesized and processed in specific cells within the pancreatic alpha cells and the intestinal L cells. In pancreatic alpha cells, proglucagon is cleaved to glucagon. However, in the intestinal L cells, proglucagon undergoes tissue-specific processing, leading to the generation of GLP-1 among other peptide fragments. This shared origin highlights the potential for overlap in the regulation and secretion of these hormones. The synthesis of both glucagon and GLP-1 from proglucagon underscores their interconnectedness and the possibility of mutual regulatory mechanisms within the endocrine system.

Moreover, glucagon and GLP-1 exhibit overlap in their effects on receptors. The immediate introduction of glucagon has been demonstrated to lessen food consumption and decrease the feeling of hunger. As a result, the observed effect of glucagon administration could be attributed in part to cross-reactivity with the GLP-1 receptor which can aid in weight loss (59) and cardiac functions.For instance, glucagon was shown to have positive inotropic and chronotropic effects (60), as well as antiarrhythmic properties (61).

Furthermore, both glucagon and GLP-1 have overlapping effects on target organs. Both glucagon and glucagon-like peptide-1 play important roles in regulating glucose and lipid metabolism, as well as appetite and food intake. It also stimulates the breakdown of fat in adipose tissue, resulting in the release of free fatty acids into the bloodstream. This leads to decreased fat mass and improved insulin sensitivity. In summary, both glucagon and GLP-1 have important roles in glucose and lipid metabolism and can contribute to a decrease in fat mass and food intake. Studies also have shown that GLP-1 and glucagon can both stimulate insulin production (62). Also, they have some overlapping effects, such as decreasing stomach peristalsis, which can additionally lead to a decrease in appetite and food intake. The administration of glucagon resulted in a significant delay in gastric emptying in all individuals who were examined. Within the first hour of glucagon infusion, the release of postprandial gastrin was inhibited, and the increase in serum gastrin concentration following a meal was delayed (63).

### Overlap in insulin and glucagon action

Both insulin and glucagon also exhibit overlap in their effects on target organs. The beta blocker overdose is treated by infusion of glucagon and insulin (high-dose insulin euglycaemic therapy – HIET) since they can independently counteract negative effects of increased beta blockage (64). The paradoxical physiological synergy of antagonistic pancreatic hormones raises the question of their potential application in heart failure. Co-administration of these hormones could potentiate each other's positive effects and possibly negate detrimental effects, similarly as they interact as an antidote to beta blocker intoxication: glucagon physiologically antagonizes BB activity via cAMP synthesis in cardiomyocytes, while insulin promote euglycaemia via activation of tyrosine kinase receptor in IR+ cells. The hormonal combinations may potentially reduce prolongation of QTc interval in T2DM (65) and fluid retention (66) caused by insulin monotherapy. The study results suggested that glucagon as an antagonistic hormone might beneficially impact cardiovascular outcomes in people with T2D treated with insulin (65), an hypothesis that needs of course be investigated in randomized controlled trials. Continuing the topic of energy expenditure, it is worth mentioning the effect of hormones on ATP production. The ATP production in muscle cells was shown to be increased by 32–42% after an 8-h infusion of high doses of insulin, basal glucagon and somatostatin (67). In this setting, somatostatin was used to reduce the secretion of the pancreatic hormones. In another study (68), the ATP/ADP ratio was increased by 30% with only glucagon infusion into the nutrient medium consisting of rat hepatocytes. The same result with glucagon was demonstrated in a study in which mitochondrial ATP was increased by 25% (69). Insulin facilitates the storage of excess glucose in the form of glycogen in the liver and muscle cells, while also promoting the synthesis of fats in adipose tissue. This process of fat synthesis is known as lipogenesis and contributes to long-term energy storage in the body. Glucagon, on the other hand, promotes energy storage in the form of glycogen in the liver when energy intake is high. This mechanism helps to regulate blood sugar levels and ensure that excess energy is stored for later use. This form of energy storage is particularly important during times of fasting or low energy intake, when the body needs to rely on stored energy reserves to maintain its metabolic functions. In summary, insulin and glucagon work together to promote the storage of energy in the body, but they do so in different forms and in response to different metabolic cues. This coordinated effort helps to maintain energy balance and ensure optimal metabolic function. It is worth mentioning the dual effect of pancreatic hormones on the regeneration of the liver tissue, consisting of insulin (IR) and glucagon receptors (GCGR) on hepatocytes. Some studies showed that the combination of insulin and glucagon regulates the regeneration of the liver. In a series of articles (70, 71, 72), the enhanced effectiveness of the two hormones increased hepatic regeneration in partially hepatectomized rats while monotherapy was not effective to such an extent. The combination of two hormones also stimulated the growth of hepatocytes in primary tissue culture (73, 74). Fiaccadori *et al.* showed that in 12 cases of fulminant hepatic failure a significant further increase of insulin and glucagon levels has been observed (75). The next study (76) showed that the limited and extended hepatectomy in a dog significantly increased insulin and glucagon levels.

Moreover, insulin and glucagon influence other physiological functions. Glucagon has significant positive effects on glucose metabolism in both fasting and postprandial states. Glucagon enhances insulin-mediated glucose control during fasting by increasing its concentration and action. This is achieved by activating both GCGR and GLP-1R on β cells, which lead to increased insulin secretion via paracrine signalling (77). On the other hand, when insulin levels remain stable in diabetes patients, an increase in glucagon leads to hyperglycaemia and glycosuria. Both insulin and glucagon play important roles in the promotion of energy storage, although they do so in distinct ways. Both insulin and glucagon can influence the rate of gastric emptying and overall GI motility, albeit in different ways. Insulin does not directly affect GI motility, but high levels of insulin, as seen in conditions like insulin resistance, can slow gastric emptying. Glucagon is known to slow gastric emptying and decrease GI motility. This slowing effect can delay the absorption of nutrients and slow the rise in blood glucose levels after eating.

Insulin and glucagon also have the ability to induce vasodilation, albeit through different mechanisms. Insulin stimulates the endothelium to produce nitric oxide, which is a powerful vasodilator. By increasing the production of nitric oxide, insulin not only enhances vasodilation but also improves blood vessel health. This effect could potentially be harnessed to treat conditions such as hypertension and atherosclerosis, which are characterized by impaired blood flow. On the other hand, glucagon indirectly causes vasodilation by inhibiting the effects of norepinephrine and angiotensin II, both of which are known vasoconstrictors. Overall, the ability of both insulin and glucagon to induce vasodilation highlights the intricate relationship between hormones and the cardiovascular system. Understanding these mechanisms could pave the way for the development of new treatments for a variety of cardiovascular conditions. Some authors claim that in diabetes patients, the addition of glucagon inhibition to standard antihyperglycaemic therapy could be a useful complementary approach (78). One interesting aspect related to glucagon's role in enhancing insulin action is the development of the bionic pancreas, which combines glucagon and insulin to prevent hypoglycaemic episodes in people with diabetes. This technology lowers average blood glucose levels in adolescents without requiring an increase in daily insulin dosage. In addition, studies have shown that a 13-h glucagon infusion in patients increases both the appearance and disappearance of glucose, indicating that the hormone's effects on human glucose metabolism extend beyond its role in increasing hepatic glucose output. These findings suggest that glucagon, which is released during fasting and the prandial response, serves to prepare metabolic tissues for the subsequent influx of nutrients that occurs during feeding (45).

This figure vividly illustrates the overlapping actions of three key hormones: insulin, GLP-1, and glucagon. It is evident that all three hormones exhibit numerous overlapping actions. Insulin and GLP-1 improve endothelial function, promote glucose uptake, increase insulin production, decrease glucagon secretion, lower glucose production, and reduce inflammation. Similarly, both glucagon and insulin promote energy storage, stimulate insulin production, delay gastric emptying, and improve hepatic regeneration. Additionally, GLP-1 and glucagon boost insulin secretion, facilitate glucose uptake, decrease feeling of hunger, decrease stomach peristalsis, induce positive inotropic and chronotropic effects, increase GLP-1 secretion, and promote lipolysis.

## Conclusion and perspectives

The spectrum of hormone overlaps (Table 1) may represent a new therapeutic paradigm that leverages the complementary action of insulin, glucagon, and GLP-1 receptor agonists. By addressing both the glycaemic control and weight management in patients with insulin-dependent diabetes and obesity, this approach could offer a more effective, comprehensive treatment strategy. However, the translation of this approach into clinical practice requires further investigation to determine the optimal dosing, timing, and administration routes, as well as to understand long-term efficacy and safety (79).

## Declaration of interest

The authors declare that there is no conflict of interest that could be perceived as prejudicing the impartiality of this review. Valdis Pirags is a senior editor of *Endocrine Connections*. Valdis Pirags was not involved in the review or editorial process for this paper, on which he is listed as an author.

## Funding

We acknowledge support from the State Research Programme project in biomedical, medical technologies, and pharmaceuticals (no. VPP-EM-BIOMEDICĪNA-2022/1-0001) and Pauls Stradiņš Clinical University Hospital Research Institute project ‘Insulīna un GLP-1 AR duāla iedarbība uz aknu un tauku metabolismu’.
